# Synthesis, Antifungal Activity and Structure-Activity Relationships of Novel 3-(Difluoromethyl)-1-methyl-1*H*-pyrazole-4-carboxylic Acid Amides

**DOI:** 10.3390/molecules20058395

**Published:** 2015-05-08

**Authors:** Shijie Du, Zaimin Tian, Dongyan Yang, Xiuyun Li, Hong Li, Changqing Jia, Chuanliang Che, Mian Wang, Zhaohai Qin

**Affiliations:** 1 Department of Applied Chemistry, College of Science, China Agricultural University, Beijing 100193, China; E-Mails: dsj5216@163.com (S.D.); yangdy@cau.edu.cn (D.Y.); lixiuyun0115@163.com (X.L.); lihong129106@163.com (H.L.); cauchangqing@gmail.com (C.J.); chechuanliang@cau.edu.cn (C.C.); woookooo@cau.edu.cn (M.W.); 2 College of Agricultural and Forestry Science and Technology, Hebei North University, Zhangjiakou 075131, Hebei, China; E-Mail: nkxtzm@163.com

**Keywords:** fungicidal activity, SDHIs, CoMFA, molecular docking

## Abstract

A series of novel 3-(difluoromethyl)-1-methyl-1*H*-pyrazole-4-carboxylic acid amides were synthesized and their activities were tested against seven phytopathogenic fungi by an *in vitro* mycelia growth inhibition assay. Most of them displayed moderate to excellent activities. Among them N-(2-(5-bromo-1*H*-indazol-1-yl)phenyl)-3-(difluoro-methyl)-1-methyl-1*H*-pyrazole-4-carboxamide (**9m**) exhibited higher antifungal activity against the seven phytopathogenic fungi than boscalid. Topomer CoMFA was employed to develop a three-dimensional quantitative structure-activity relationship model for the compounds. In molecular docking, the carbonyl oxygen atom of **9m** could form hydrogen bonds towards the hydroxyl of TYR58 and TRP173 on SDH.

## 1. Introduction

**Figure 1:**
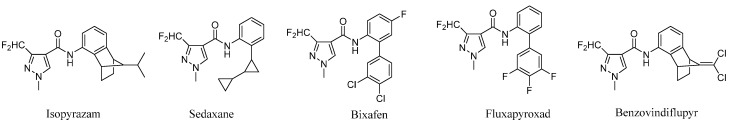
Commercial available 3-(difluoromethyl)-1-methyl-1*H*-pyrazole-4-carboxylic acid amide fungicides.

Amide compounds are traditional fungicides. Their common target is mitochondrial respiratory chain enzyme complex II (succinate dehydrogenase, SDH) [1]. With the development of boscalid, many researchers paid attention to the amide fungicides with a renewed focus on this traditional fungicide class. A series of novel highly efficient amide fungicides have been used for crop protection. In particular, the 3-(difluoromethyl)-1-methyl-1*H*-pyrazole-4-acyl group has been the most outstanding acyl moiety group in recent years, and a number of excellent commercial fungicides with this group were successfully developed, such as: isopyrazam (Syngenta, 2010), sedaxane (Syngenta, 2011), bixafen (Bayer, 2011), fluxapyroxad (BASF, 2012) and benzovindiflupyr (Syngenta, 2012) (Figure 1).

**Scheme 1:**
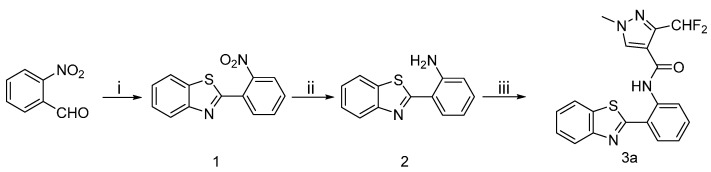
Synthetic procedure for target compounds **3a**.

**Scheme 2:**
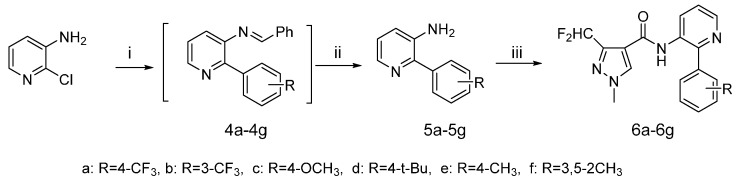
Synthetic procedure for target compounds **5a**–**5g**.

**Scheme 3:**
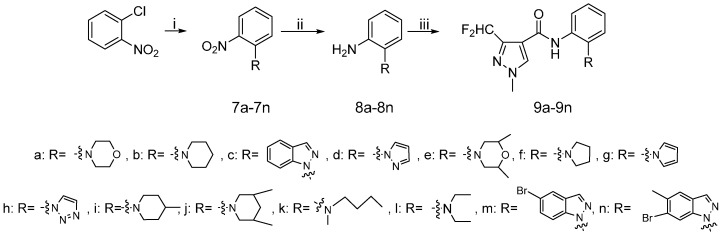
Synthetic procedure for target compounds **9a**–**9n**.

In our previous work [2], we designed and synthesized a series of amide compounds based on bioisosterism by introducing an N atom instead the C atom in an *ortho*-aniline. Bioassays showed that some target molecules exhibited excellent antifungal activity against *Pythium aphanidermatum* and *Rhizoctonia solani*. By summarizing the structural characteristics of the recent commercial pyrazole amide fungicides and the previous experience in our group, we designed new some molecules according to the active fragments mosaic theory and introduced the 3-(difluoromethyl)-1-methyl-1*H*-pyrazole-4-carboxylic acyl group into these substituted anilines. Three series of novel amides were thus synthesized as shown in Scheme 1, Scheme 2 and Scheme 3. Some target molecules exhibited good antifungal activity. We report these results in this paper.

## 2. Results and Discussion

### 2.1. Synthesis of Compounds

The synthetic route to the target compounds **3a** is shown in Scheme 1. The aldehyde in 2-nitrobenzaldehyde and amine in 2-aminobenzenethiol were condensed to form an imine, then the intermediate **1** was obtained via a intramolecular cyclization between the thiol and imine. The nitro group of **1** was reduced with hydrazine hydrate to provide the intermediates **2**, which were subsequently acylated to produce the target amides **3a**.

The synthetic route to the target compounds **6a**–**6g** is shown in Scheme 2. First, the aminopyridine was protected as the corresponding benzaldehyde imine, so the formation could be accomplished *in situ* under the cross-coupling conditions. The intermediates **4** were prepared via a Suzuki coupling by reacting the imines and phenylboronic acid under palladium catalysis. Intermediates **5** were obtained after subsequent cleavage of the imines using HCl.

*Reagents and conditions*: (i) K_3_[Fe(CN)_6_], 2-aminobenzenethiol, toluene, reflux, 10 h; (ii) NH_2_-NH_2_ H_2_O, Pd-C, CH_3_OH, reflux, 8 h; (iii) **10**, TEA, CH_2_Cl_2_, 0 °C, 30 min.

*Reagents and conditions*: (i) phenylboronic acid, benzaldehyde, Pd (PPh_3_)_2_Cl_2_, Na_2_CO_3_, toluene,7 h; (ii) HCl; (iii) **10**, TEA, CH_2_Cl_2_, 0 °C, 30 min.

The synthetic route to the target compounds **9a**–**9n** is shown in Scheme 3. The chlorine atom in 1-chloro-2-nitrobenzene was replaced by an amino group via an aromatic nucleophilic substitution reaction, giving the intermediates **7**. Then the reduction of the nitro group and the acylation of the amino group were performed exactly as presented in Scheme 1.

*Reagents and conditions*: (i) H-R, HMTA, CuI, Cs_2_CO_3_, DMF, 20h; (ii) NH_2_-NH_2_ H_2_O, Pd-C, CH_3_OH, reflux, 8 h; (iii) **10**, TEA, CH_2_Cl_2_, 0 °C, 30 min.

**Scheme 4:**
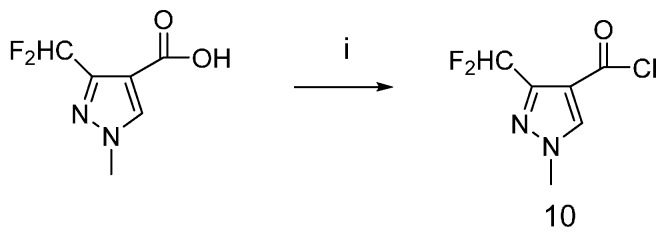
Synthetic procedure for carboxylic acid chloride **10**.

The synthetic route to the carboxylic acid chloride **10** is shown in Scheme 4. The carboxylic acid was treated with thionyl chloride, then the carboxylic acid chloride was obtained.

*Reagents and conditions*: (i) SOCl_2_, toluene, DMF, 2 h.

### 2.2. In Vitro Antifungal Activity

The *in vivo* antifungal activity results of the title compounds against seven phytopathogenic fungi are listed in Table 1. 

**Table 1:** *In vitro* antifungal activity of the target compounds against seven phyto-pathogenic fungi (50 μg·mL^−1^).

No.	Inhibition Rate (%)						
	C.O.	R.S.	P.I.	P.A.	F.S.	B.B.	B.C.
**3a**	35.29	37.32	26.77	26.15	24.00	41.48	33.90
**6a**	29.41	53.56	22.73	37.67	24.31	67.52	37.07
**6b**	83.66	44.73	26.77	61.38	17.24	50.86	69.75
**6c**	56.21	31.91	36.87	48.85	22.47	32.11	48.05
**6d**	48.37	60.11	8.59	34.51	25.24	88.92	49.27
**6e**	69.28	39.60	28.79	77.24	29.85	61.65	63.90
**6f**	53.59	38.46	29.80	43.90	27.08	45.17	51.70
**9a**	79.74	42.74	51.52	55.28	22.47	53.13	55.12
**9b**	84.97	54.13	53.03	73.98	40.31	69.89	97.56
**9c**	92.81	92.59	56.06	90.79	39.28	83.24	69.27
**9d**	54.90	35.62	30.30	45.26	26.77	48.87	37.56
**9e**	56.21	47.29	51.01	42.82	26.16	70.46	79.02
**9f**	62.75	95.16	35.86	77.51	26.16	66.20	84.88
**9g**	48.37	70.66	47.98	48.78	24.00	60.8	76.58
**9h**	25.49	51.28	60.10	27.10	43.39	33.81	37.80
**9i**	83.63	45.58	40.91	81.30	22.47	61.37	78.78
**9j**	74.51	56.41	51.52	53.66	26.16	79.83	81.46
**9k**	78.43	82.62	72.73	62.33	35.39	51.71	68.05
**9l**	52.29	88.89	56.06	46.25	41.54	35.52	49.75
**9m**	94.12	86.32	51.52	90.24	38.47	84.38	86.10
**9n**	84.97	76.07	42.93	65.67	12.01	68.18	55.36
Boscalid	83.61	91.74	36.36	85.64	31.08	79.55	83.66

C.O. = *Colletotrichum orbiculare*; R.S. = *Rhizoctonia solani*; P.I. = *Phytophthora infestans* (Mont.) De Bary; P.A. = *Pythium aphanidermatum*; F.S. = *Fusarium moniliforme Sheld*; B.B. = *Botryosphaeria berengeriana*; B.C. = *Botrytis cinerea*.

The target molecules exhibited different levels of antifungal activity against these fungi. Their inhibitory activities to *Colletotrichum orbiculare*, *Rhizoctonia solani*, *Pythium aphanide- rmatum* and *Botrytis cinerea* were higher than against the other three fungi.

Compound **9h** exhibited excellent activity against *Fusarium moniliforme Sheld* and *Rhizoctonia solani*, but little effect on the others. Among compounds **9a**–**9n**, the compounds with an indazole group (**9m** and **9n**) exhibited the highest antifungal activity against the tested fungi. The second group are the compounds with open chain tertiary amines (**9k** and **9l**) and the third one are the amides with nitrogen-containing aliphatic rings (**9a**, **9b**, *etc.*). Those compounds with imidazole or triazole rings (**9d** and **9h**) exhibited little antifungal activity. The results show the compounds **9c** and **9m** exhibited higher activity against most of the seven fungi than boscalid and the series **3** and series **6** displayed poorer activities than the series **9**.

In order to further study the activities of the best target compounds, we chose five compounds for precise virulence measurements against the seven fungi. Table 2 shows their EC_50_ values. As we can see from Table 2, compound **9m** displayed excellent activity against *Colletotrichum orbiculare*, *Rhizoctonia solani*, *Phytophthora infestans* (Mont.) De Bary, *Fusarium moniliforme Sheld* and *Botryosphaeria berengeriana* with EC_50_ values of 5.50, 14.40, 75.54, 79.42 and 28.29, respectively. It thus exhibited lower EC_50_ values and a broader spectrum of antifungal activity than the control boscalid.

**Table 2:** EC_50_ values of target compounds against seven fungi (μg·mL^−1^).

No.	C.O.	R.S.	P.I.	P.A.	F.S.	B.B.	B.C.
**9b**	33.99	55.83	80.94	35.35	96.21	29.13	24.87
**9c**	21.03	26.92	86.85	31.65	83.52	28.71	32.80
**9f**	26.71	16.55	94.24	30.84	86.57	46.29	20.72
**9k**	25.94	21.92	39.22	26.12	58.17	49.22	41.35
**9m**	5.50	14.40	75.54	21.04	79.42	28.29	30.69
Boscalid	5.86	15.48	79.80	15.58	86.28	33.39	19.69

### 2.3. QSAR Analyses

The topomer CoMFA model was optimized. A cross-validation q^2^ value of 0.636 and a non-cross-validation r^2^ value of 0.995 with an optimized component of 6 were obtained, which suggested that the model has good predictive ability (q^2^ > 0.5). The sterically favored and disfavored regions are shown in green and yellow. In the electrostatic field, the positively charged favored regions are shown in blue, and the negatively charge favored regions are shown in red.

**Figure 2:**
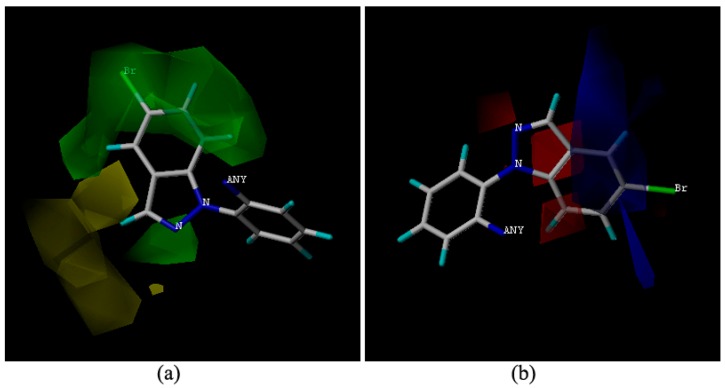
Topomer CoMFA contour maps around the amine moiety. (**a**) steric field around the amine moiety of **9m**; (**b**) electrostatic field around the amine moiety of **9m**.

We chose the molecule **9m** with highest activity which makes it is easier to explain the contour map. The bromoindazole group in compound **9m**, with an increasing steric hindrance with its larger substituent exhibits stronger bioactivities in the steric field map (Figure 2a). The compound **9h** with a small triazole group exhibited poor activity. In the electrostatic region, the benzene ring of the indazole hovered in the blue blocks (Figure 2b), indicating that electropositivity was beneficial for the antifungal activity. Compound **9m** has a more electropositive benzene ring which may be attributed to that the N atoms in the indazole which decrease the electron density of the benzene ring.

### 2.4. Molecular Docking

**Figure 3:**
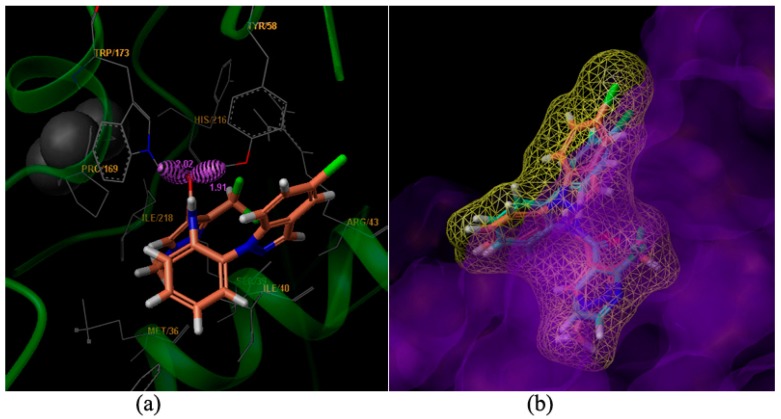
Surflex-Docking of compound **9m** to complex II. (**a**) Interaction of **9m** and amino acid residues near the ligands (3D diagram); (**b**) Connolly surface of complex II with compound **9m** and boscalid shown as a stick model.

In an effort to elucidate the possible mechanism of the observed antifungal activity of these compounds, molecular docking of compounds **9m** to the binding site of SDH (pdb code: 2FBW [3,4]) pdb was performed. The three-dimensional schematic diagrams clearly explained the possible optimal combination between the ligands and receptor protein (Figure 3).

Compound **9m** is bound to the Q_p_ [5] site of SDH. Two hydrogen bonds were formed between the carboxyl oxygen of compound **9m** and amino acid residues. The hydrogen bonding distance between the amino hydrogen of TRP173 and the carboxyl oxygen of **9m** was found to be 2.02 Å. Another hydrogen bond between the hydroxyl hydrogen of TYR58 and the carboxyl oxygen of **9m** was found to be 1.91 Å (Figure 3a). The amino acid residues of MET36, SER39, ILE40, AGR43, PRO169, HIS216 and ILE218 interacted with the ligand, including weak interaction such as wan der Waals interactions and polar interactions. The results agreed well with the molecule docking of carboxin [6], boscalid and bixafen [7].

The compounds **9m** (the light salmon color) and boscalid (the light blue color) were aligned in the active pocket (Figure 3b). The total docking scores were 6.59 and 5.26, respectively. The carboxylic acid building block moiety appears to be more harmonious than the amide building block. The results of this molecular docking study demonstrated the perfect combination between compound **9m** and the key residues in the binding cavity of SDH [8]. Thus, the stable complex could support the postulation that our active compounds may act on the same enzyme target where SDH inhibitors act confirming the molecular design of the reported class of antifungal agents.

## 3. Experimental Section

### 3.1. General Information

^1^H (300 MHz) and ^13^C-NMR (75 MHz) spectra were obtained using an Avance DPX300 spectrometer (Bruker, Billerica, MA, USA) in CDCl_3_ or DMSO-*d*_6_ solution with tetramethylsilane as the internal standard. Melting points were determined using an X-4 binocular microscope melting point apparatus (Beijing Tech Instruments Co., Beijing, China). High resolution mass spectrometry data were obtained with an Accurate-Mass-Q-TOF MS 6520 system equipped with an electrospray ionization (ESI) source (Agilent, Santa Clara, CA, USA).

### 3.2. Synthesis of Compounds

#### 3.2.1. Synthesis of 2-(2-Nitrophenyl)benzo[*d*]thiazole (**1**)

In a 250 mL flask, 2-aminobenzenethiol (5.7 g, 45.9 mmol), 2-nitrobenzaldehyde (22.6 g, 68.7 mmol), and K_3_[Fe(CN)_6_] (22.7 g, 6.9 mmol) were dispersed in toluene (100 mL), and the mixture was heated to reflux for 10 h [9]. Product **1** was obtained by the filtration of the reaction mixture, concentration of the filtrate and recrystallization of the crude product from ethanol. Orange powder; yield: 8.5 g (72.2%); mp 126–127 °C. ^1^H-NMR (CDCl_3_) δ 8.08 (m, 1H), 8.01–7.87 (m, 2H), 7.79 (m, 1H), 7.75–7.59 (m, 2H), 7.59–7.49 (m, 1H), 7.45 (m, 1H).

#### 3.2.2. Synthesis of 2-(Benzo[*d*]thiazol-2-yl)anilines **2** and **8a**–**8m**

In a 100 mL three-necked flask equipped with a dropping funnel, 2-(2-nitrophenyl)benzo[d]- thiazole (**1**, 3.0 g, 12.5 mmol) and ethanol (50 mL) were mixed and heated to reflux. Palladized charcoal (0.1 g, 5%) was added, then 80% hydrazine hydrate solution (10 mL) was added from a dropping funnel during 30 min [10,11]. The heating was continued for 8 h and then the mixture cooled. The solid was filtered off and the filtrate was concentrated to give a crude product that was recrystallized from ethyl acetate and petroleum ether solution (1:5) to afford compound **2** as a yellow powder (yield: 3.76 g, 85%); mp 130–131 °C. ^1^H-NMR (CDCl_3_) δ 7.97 (dd, *J* = 8.1, 0.6 Hz, 1H), 7.92–7.82 (m, 1H), 7.71 (dd, *J* = 7.9, 1.1 Hz, 1H), 7.53–7.41 (m, 1H), 7.39–7.31 (m, 1H), 7.25–7.16 (m, 1H), 6.84–6.68 (m, 2H), 6.40 (s, 2H). Compounds **8a**–**8m** were similarly prepared from **7a**–**7m**.

#### 3.2.3. General Procedure for the Preparation of 2-Aryl-3-aminopyridines **5a**–**5g**

In a 250 mL flask, 2-chloropyridin-3-amine (5 g, 38.9 mmol), *p*-tolylboronic acid (5.5 g, 46.7 mmol), benzaldehyde (4.12 g, 38.9 mmol) and toluene (80 mL) were stirred at room temperature for 10 min, and *trans*-dichloro(triphenylphosphine) palladium [5] (0.136 g, 0.21 mmol) was added. The mixture was stirred for 15 min, and a solution of Na_2_CO_3_ (5.0 g, 46.7mmol) in water (80 mL) was added. The mixture was heated to reflux for 7 h. The suspension was filtered when the mixture was cooled to room temperature, and the layers were allowed to separate. The organic layer was treated with 3 N HCl (60 mL). The organic layer containing the benzaldehyde was discarded and the pH adjusted to 12 with 50% aqueous NaOH [12]. After extraction with ethyl acetate (3×60 mL) the organic layer was concentrated to an oil. Pure 2-(*p*-tolyl)pyridin-3-amine (**5f**) was obtained as a brown paste by column chromatography (EtOAc-PE = 4:1) purification. Yield: 5.20 g (72%); ^1^H-NMR (CDCl_3_) δ 8.01 (dd, *J* = 7.4, 1.5 Hz, 1H), 7.98 (d, *J* = 7.5 Hz, 2H), 7.15 (d, *J* = 7.5 Hz, 2H), 6.94 (t, *J* = 7.5 Hz, 1H), 6.82 (dd, *J* = 7.5, 1.4 Hz, 1H), 3.79 (s, 2H), 2.34 (s, 3H).

#### 3.2.4. General Procedures for the Preparation of 2-Substituted Aminoanilines **3a**, **6a**–**6g** and **9a**–**9n**

These reactions were performed as described in [13] and [14].

*N-(2-(Benzo[d]thiazol-2-yl)phenyl)-3-(difluoromethyl)-1-methyl-1H-pyrazole-4-carboxamide* (**3a**)*.* White crystals; yield 70%; mp 212–214 °C; ^1^H-NMR (CDCl_3_) δ 12.76 (s, 1H, NH), 8.94–8.69 (m, 1H, Ar-H), 8.08 (s, 1H, pyrazole-H), 7.93 (d, *J* = 7.8 Hz, 1H, Ar-H), 7.88–7.76 (m, 2H, Ar-H), 7.59–7.49 (m, 1H, Ar-H), 7.50–7.40 (m, 2H, Ar-H), 7.32 (t, 1H, CF_2_H), 7.19–7.06 (m, 1H, Ar-H), 4.09 (s, 3H, CH_3_). ^13^C-NMR (CDCl_3_) δ 160.03, 152.40, 137.72, 133.19, 132.02, 130.93, 129.71, 126.60, 125.75, 123.19, 121.62, 121.34, 120.63, 109.22, 39.67. HRMS (ESI), *m/z* calcd for C_19_H_14_F_2_N_4_OS (M+H)^+^ 385.0929, found 385.0930.

*3-(Difluoromethyl)-1-methyl-N-(2-(4-(trifluoromethyl)phenyl)pyridin-3-yl)-1H-pyrazole-4-carboxamide* (**6a**). White powder; yield 77%; mp 170–172 °C; ^1^H-NMR (CDCl_3_) δ 8.81 (dd, *J* = 8.2, 1.7 Hz, 1H, pyridine-H), 8.54 (s, 1H, NH), 8.15 (dd, *J* = 4.7, 1.7 Hz, 1H, pyridine-H), 8.05 (s, 1H, pyrazole-H), 7.86–7.76 (m, 1H, Ar-H), 7.39–7.25 (m, 3H, Ar-H), 7.07–7.02 (m, 1H, pyridine-H), 6.83 (t, 1H, CF_2_H), 3.96 (s, 3H). ^13^C-NMR (CDCl_3_) δ 159.43, 143.88, 140.22, 138.47, 135.58, 131.93, 129.36, 123.11, 122.92, 122.15, 116.20, 111.26, 60.16, 39.47, 13.98. HRMS (ESI), *m/z* calcd for C_18_H_13_F_5_N_4_O (M+H)^+^ 397.1082, found 397.1083.

*3-(Difluoromethyl)-1-methyl-N-(2-(3-(trifluoromethyl)phenyl)pyridin-3-yl)-1H-pyrazole-4-carboxamide* (**6b**): White crystals; yield 68%; mp 152–154 °C; ^1^H-NMR (CDCl_3_) δ 8.52 (dd, *J* = 8.3, 1.3 Hz, 1H, pyridine-H), 8.44 (dd, *J* = 4.7, 1.4 Hz, 1H, pyridine-H), 8.13–7.92 (s, 1H, NH), 7.84 (s, 1H, pyrazole-H) 7.82 (d, 1H, Ar-H), 7.63 (ddd, *J* = 29.9, 19.5, 11.0 Hz, 3H, Ar-H), 7.32 (dd, *J* = 8.3, 4.7 Hz, 1H, pyridine-H), 6.71 (t, 1H, CF_2_H), 3.86 (s, 3H, CH_3_). ^13^C-NMR (CDCl_3_) δ 159.57, 149.00, 145.57, 137.96, 135.06, 132.19, 131.65, 130.81, 129.30, 125.61, 125.55, 125.52, 125.45, 125.40, 123.12, 115.90, 110.85, 39.31. HRMS (ESI), *m/z* calcd for C_18_H_13_F_5_N_4_O (M+H)^+^ 397.1082, found 397.1080.

*3-(Difluoromethyl)-N-(2-(4-methoxyphenyl)pyridin-3-yl)-1-methyl-1H-pyrazole-4-carboxamide* (**6c**): White crystals; yield 75%; mp 188–190 °C; ^1^H-NMR (CDCl_3_) δ 8.45 (dd, *J* = 8.3, 1.4 Hz, 1H, pyridine-H), 8.34 (dd, *J* = 4.7, 1.5 Hz, 1H, pyridine-H), 8.10 (s, 1H, NH), 7.73 (s, 1H, pyrazole-H), 7.51–7.40 (m, 2H, Ar-H), 7.21 (dd, *J* = 8.3, 4.7 Hz, 1H, pyridine-H), 6.99–6.89 (m, 2H, Ar-H), 6.86 6.71 (t, 1H,CF_2_H), 3.82 (s, 3H, CH_3_)3.80 (s, 3H, CH_3_). ^13^C-NMR (CDCl_3_) δ 159.96, 159.60, 149.98, 145.18, 133.98, 131.36, 130.00, 129.37, 122.10, 114.19, 110.45, 55.16, 39.37. HRMS (ESI), *m/z* calcd for C_18_H_16_F_2_N_2_O_2_ (M+H)^+^ 359.1314, found 359.1315.

*N-(2-(4-(tert-Butyl)phenyl)pyridin-3-yl)-3-(difluoromethyl)-1-methyl-1H-pyrazole-4-carboxamide* (**6d**): White crystals; yield 73%; mp 178–180 °C; ^1^H-NMR (CDCl_3_) δ 8.59 (dd, *J* = 8.3, 1.4 Hz, 1H, pyridine-H), 8.39 (dd, *J* = 4.7, 1.5 Hz, 1H, pyridine-H), 8.03 (s, 1H, NH), 7.75 (s, 1H, pyrazole-H), 7.58–7.35 (m, 4H, Ar-H), 7.26 (dd, *J* = 8.2, 4.8 Hz, 1H, pyridine-H), 6.74 (t, 1H, CF_2_H), 3.87 (s, 3H, CH_3_), 1.34 (s, 9H, CH_3_). ^13^C-NMR (CDCl_3_) δ 159.51, 151.94, 150.11, 145.05, 134.29, 134.00, 131.60, 129.44, 128.30, 125.78, 122.32, 116.45, 110.43, 39.38, 34.54, 31.04. HRMS (ESI), *m/z* calcd for C_21_H_22_F_2_N_4_O (M+H)^+^ 385.1834, found 385.1835.

*3-(Difluoromethyl)-1-methyl-N-(2-(p-tolyl)pyridin-3-yl)-1H-pyrazole-4-carboxamide* (**6e**): Yellow crystals; yield 75%; mp 168–170 °C; ^1^H-NMR (CDCl_3_) δ 8.55 (dd, *J* = 8.3, 1.4 Hz, 1H, pyridine-H), 8.39 (dd, *J* = 4.7, 1.4 Hz, 1H, pyridine-H), 8.05 (s, 1H, NH), 7.75 (s, 1H, pyrazole-H), 7.41 (d, *J* = 8.0 Hz, 2H, Ar-H), 7.26 (d, 2H, Ar-H), 7.25 (dd, *J* = 8.2, 4.8 Hz, 1H, pyridine-H) 6.80(t, 1H, CF_2_H), 3.89 (s, 3H, CH_3_), 2.39 (s, 3H, CH_3_). ^13^C-NMR (CDCl_3_) δ 159.58, 150.16, 145.13, 138.71, 134.09, 133.96, 131.46, 129.78, 129.44, 128.49, 122.28, 116.36, 110.42, 39.37, 21.10. HRMS (ESI), *m/z* calcd for C_18_H_16_F_2_N_4_O (M+H)^+^ 343.1365, found 343.1365.

*3-(Difluoromethyl)-N-(2-(3,5-dimethylphenyl)pyridin-3-yl)-1-methyl-1H-pyrazole-4-carboxamide* (**6f**): White crystals; yield 71%; mp 198–200 °C; ^1^H-NMR (CDCl_3_) δ 8.55 (dd, *J* = 8.3, 1.3 Hz, 1H, pyridine-H), 8.34 (dd, *J* = 4.7, 1.4 Hz, 1H, pyridine-H), 8.08 (s, 1H, NH), 7.72 (s, 1H, pyrazole-H), 7.33–7.16 (m, 1H, pyridine-H), 7.06 (d, *J* = 14.9 Hz, 3H, Ar-H), 6.79 (t, 1H, CF_2_H), 3.84 (s, 3H, CH_3_), 2.30 (s, 6H, CH_3_). ^13^C-NMR (CDCl_3_) δ 159.57, 150.40, 144.93, 143.63, 138.39, 136.84, 133.99, 131.49, 130.35, 129.64, 126.25, 122.27, 116.31, 110.39, 39.31, 21.02. HRMS (ESI), *m/z* calcd for C_19_H_18_F_2_N_4_O (M+H)^+^ 357.1521, found 357.1521.

*3-(Difluoromethyl)-1-methyl-N-(2-morpholinophenyl)-1H-pyrazole-4-carboxamide* (**9a**). Brown powder; yield 70%; mp 174–176 °C; ^1^H-NMR (CDCl_3_) δ 9.28 (s, 1H, NH), 8.43 (dd, *J* = 8.1, 1.5 Hz, 1H, Ar-H), 7.97 (s, 1H, pyrazole-H), 7.36–7.10 (m, 3H, Ar-H), 7.09–6.74 (t, 1H, CF_2_H), 3.95 (s, 3H, CH_3_), 3.90–3.77 (m, 4H, CH_2_), 3.02–2.72 (m, 4H, CH_2_). ^13^C-NMR (CDCl_3_) δ 159.19, 141.47, 134.83, 133.78, 125.66, 124.05, 121.11, 120.21, 117.39, 111.18, 66.89, 52.68, 39.39. HRMS (ESI), *m/z* calcd for C_16_H_18_F_2_N_4_O_2_ (M+H)^+^ 337.1471, found 337.1469.

*3-(Difluoromethyl)-1-methyl-N-(2-(piperidin-1-yl)phenyl)-1H-pyrazole-4-carboxamide* (**9b**): Orange powder; yield 72%; mp 88–90 °C; ^1^H-NMR (CDCl_3_) δ 9.08 (s, 1H, NH), 8.28 (dd, *J* = 7.9, 1.6 Hz, 1H, Ar-H), 7.81 (s, 1H, pyrazole-H), 7.13–6.89 (m, 3H, Ar-H), 6.85 (t, 1H, CF_2_H), 3.82 (s, 3H, CH_3_), 2.77–2.52 (m, 4H, CH_2_), 1.69–1.53 (m, 4H, CH_2_), 1.47 (d, *J* = 4.7 Hz, 2H, CH_2_). ^13^C-NMR (CDCl_3_) δ 159.22, 133.42, 133.10, 124.94, 123.82, 120.71, 119.70, 110.41, 60.15, 53.83, 39.46, 26.38, 23.80, 13.98. HRMS (ESI), *m/z* calcd for C_17_H_20_F_2_N_2_O (M+H)^+^ 335.1678, found 335.1677.

*N-(2-(1H-Indazol-1-yl)phenyl)-3-(difluoromethyl)-1-methyl-1H-pyrazole-4-carboxamide* (**9c**): White crystals; yield 69%; mp 150–152 °C; ^1^H-NMR (CDCl_3_) δ 9.74 (s, 1H, NH), 8.50 (dd, *J* = 8.3, 1.3 Hz, 1H, Ar-H), 8.32 (d, *J* = 0.7 Hz, 1H, Ar-H), 7.81 (d, *J* = 8.1 Hz, 1H, Ar-H), 7.67 (s, 1H, pyrazole-H), 7.62–7.48 (m, 2H, Ar-H), 7.48–7.36 (m, 2H, Ar-H), 7.35–7.20 (m, 2H, Ar-H), 7.21 (t, 1H, CF_2_H), 3.89 (s, 3H, CH_3_). ^13^C-NMR (CDCl_3_) δ 159.24, 139.84, 135.75, 132.28, 131.55, 128.61, 128.11, 127.82, 124.32, 124.25, 124.22, 123.36, 122.03, 121.16, 116.90, 110.53, 109.57, 39.47. HRMS (ESI), *m/z* calcd for C_19_H_15_F_2_N_5_O (M+H)^+^ 368.1317, found 368.1323.

*N-(2-(1H-Pyrazol-1-yl)phenyl)-3-(difluoromethyl)-1-methyl-1H-pyrazole-4-carboxamide* (**9d**): White crystals; yield 67%; mp 152–154 °C; ^1^H-NMR (CDCl_3_) δ 10.98 (s, 1H, NH), 8.76–8.29 (m, 1H, Ar-H), 7.84 (dd, *J* = 5.7, 2.1 Hz, 2H, pyrazole-H), 7.76 (s, 1H, pyrazole-H), 7.39–7.29 (m, 2H, Ar-H), 7.28 (t, 1H, CF_2_H), 7.14 (d, 1H, Ar-H), 6.47 (m, 1H, pyrazole-H), 3.96 (s, 3H, CH_3_). ^13^C-NMR (CDCl_3_) δ 159.36, 140.81, 130.97, 130.95, 130.14, 128.59, 127.72, 124.00, 122.66, 121.89, 109.36, 107.17, 39.57. HRMS (ESI), *m/z* calcd for C_15_H_13_F_2_N_5_O (M+H)^+^ 318.1161, found 318.1161.

*3-(Difluoromethyl)-N-(2-(2,6-dimethylmorpholino)phenyl)-1-methyl-1H-pyrazole-4-carboxamide* (**9e**): White crystals; yield 72%; mp 182–184 °C; ^1^H-NMR (CDCl_3_) δ 9.25 (s, 1H, NH), 8.53–8.31 (m, 1H, Ar-H), 7.92 (s, 1H, pyrazole-H), 7.21–7.01 (m, 3H, Ar-H), 6.88 (t, 1H, CF_2_H), 3.90 (s, 3H, CH_3_), 3.83 (dd, *J* = 13.8, 6.9 Hz, 2H, CH_2_), 2.75 (d, *J* = 10.9 Hz, 2H, CH_2_), 2.46 (t, *J* = 10.8 Hz, 2H, CH_2_), 1.16 (d, *J* = 6.3 Hz, 6H, CH_3_). ^13^C-NMR (CDCl_3_) δ 159.18, 141.14, 134.34, 133.80, 125.54, 123.96, 121.18, 119.98, 117.31, 110.97, 71.68, 58.27, 39.35, 18.81. HRMS (ESI), *m/z* calcd for C_18_H_12_F_2_N_4_O_2_ (M+H)^+^ 365.1784, found 365.1789.

*3-(Difluoromethyl)-1-methyl-N-(2-(pyrrolidin-1-yl)phenyl)-1H-pyrazole-4-carboxamide* (**9f**): White powder; yield 75%; mp 134–136 °C; ^1^H-NMR (300 MHz, CDCl_3_) δ 9.03 (s, 1H,NH), 8.17 (t, *J* = 10.4 Hz, 1H, Ar-H), 7.92 (s, 1H, pyrazole-H), 7.22–6.93 (m, 3H), 7.10 (t, 1H, CF_2_H), 3.87 (s, 3H, CH_3_), 3.00 (d, *J* = 5.6 Hz, 4H, CH_2_), 1.96 (d, *J* = 34.0 Hz, 4H, CH_2_). ^13^C-NMR (CDCl_3_) δ 159.38, 141.31, 134.38, 124.55, 123.24, 121.49, 119.40, 117.20, 114.00, 110.89, 107.79, 52.28, 39.28, 24.32. HRMS (ESI), *m/z* calcd for C_16_H_18_F_2_N_4_O (M+H)^+^ 321.1521, found 321.1525.

*N-(2-(1H-Pyrrol-1-yl)phenyl)-3-(difluoromethyl)-1-methyl-1H-pyrazole-4-carboxamide* (**9g**): Light yellow powder; yield 79%; mp 146–148 °C; ^1^H-NMR (CDCl_3_) δ 8.37 (dd, *J* = 8.3, 1.3 Hz, 1H, Ar-H), 7.57 (s, 1H, NH), 7.55(s, 1H, pyrazole-H), 7.44–7.35 (m, 1H, Ar-H), 7.30 (dt, *J* = 8.6, 4.3 Hz, 1H, Ar-H), 7.19 (dt, *J* = 7.7, 1.2 Hz, 1H, Ar-H), 6.98 (t, 1H, CF_2_H), 6.82 (t, *J* = 2.1 Hz, 2H, pyrrole-H), 6.41 (t, *J* = 2.1 Hz, 2H, pyrrole-H), 3.86 (s, 3H, CH_3_). ^13^C-NMR (CDCl_3_) δ 159.27, 133.39, 132.51, 131.09, 128.55, 126.79, 124.39, 122.05, 121.76, 116.44, 110.23, 109.93, 39.46. HRMS (ESI), *m/z* calcd for C_16_H_14_F_2_N_4_O (M+H)^+^ 317.1208, found 317.1209.

*N-(2-(1H-1,2,3-Triazol-1-yl)phenyl)-3-(difluoromethyl)-1-methyl-1H-pyrazole-4-carboxamide* (**9h**): Light yellow powder; yield 62%; mp 188–190 °C; ^1^H-NMR (DMSO-*d_6_*) δ 10.49 (s, 1H, NH), 8.34 (s, 1H, pyrazole-H), 8.17 (m, 2H, Ar-H), 8.11–8.01 (m, 1H, triazole-H), 7.88 (m, 1H, triazole-H), 7.43 (dd, 2H, Ar-H), 7.26 (t, 1H, CF_2_H), 3.98 (s, 3H, CH_3_). ^13^C-NMR (DMSO-*d_6_*) δ 159.88, 145.18, 136.31, 132.99, 131.29, 129.99, 128.78, 125.62, 125.34, 123.70, 116.30, 109.89, 39.73. HRMS (ESI), *m/z* calcd for C_14_H_12_F_2_N_6_O (M+H)^+^ 319.1113, found 319.1112.

*3-(Difluoromethyl)-1-methyl-N-(2-(4-methylpiperidin-1-yl)phenyl)-1H-pyrazole-4-carboxamide* (**9i**): White crystals; yield 76%; mp 137–139 °C; ^1^H-NMR (CDCl_3_) δ 9.17 (s, 1H, NH), 8.37 (dd, *J* = 7.9, 1.5 Hz, 1H, Ar-H), 7.87 (s, 1H, pyrazole-H), 7.31–6.84 (m, 3H, Ar-H), 7.08 (t, 1H, CF_2_H), 3.91 (s, 3H, CH_3_), 3.01–2.80 (m, 2H, CH_2_), 2.66 (dd, *J* = 16.6, 6.6 Hz, 2H, CH_2_), 1.72 (d, *J* = 12.3 Hz, 2H, CH_2_), 1.49 (dd, *J* = 10.6, 6.2 Hz, 1H, CH), 1.33 (tdd, *J* = 19.8, 14.1, 5.7 Hz, 2H, CH_2_), 0.98 (d, *J* = 6.3 Hz, 3H,CH_3_). ^13^C-NMR (CDCl_3_) δ 159.22, 143.99, 142.80, 133.41, 133.12, 124.79, 123.80, 120.70, 119.69, 117.46, 110.45, 53.11, 39.39, 34.74, 30.23, 21.75. HRMS (ESI), *m/z* calcd for C_18_H_22_F_2_N_4_O (M+H)^+^ 349.1834, found 349.1838.

*3-(Difluoromethyl)-N-(2-(3,5-dimethylpiperidin-1-yl)phenyl)-1-methyl-1H-pyrazole-4-carboxamide* (**9j**): Yellow oily liquid; yield 75%; ^1^H-NMR (CDCl_3_) δ 9.18 (s, 1H, NH), 8.38 (dd, *J* = 8.0, 1.5 Hz, 1H, Ar-H), 7.81 (s, 1H, pyrazole-H), 7.22–6.94 (m, 3H, Ar-H), 7.10 (t, 1H, CF_2_H), 3.91 (s, 3H, CH_3_), 2.91–2.76 (m, 2H, CH_2_), 2.30–2.10 (m, 2H, CH_2_), 1.94–1.71 (m, 2H, CH_2_), 1.00 (d, *J* = 6.8 Hz, 1H, CH), 0.86 (d, *J* = 6.4 Hz, 6H, CH_3_), 0.75–0.59 (m, 1H, CH). ^13^C-NMR (CDCl_3_) δ 159.21, 142.42, 133.48, 132.76, 124.92, 123.77, 120.84, 119.55, 117.46, 110.3, 60.56, 41.68, 39.41, 31.77, 19.14. HRMS (ESI), *m/z* calcd for C_19_H_24_F_2_N_2_O (M+H)^+^ 363.1991, found 363.1990.

*N-(2-(Butyl(methyl)amino)phenyl)-3-(difluoromethyl)-1-methyl-1H-pyrazole-4-carboxamide* (**9k**): Yellow crystals; yield 78%; mp 18–20 °C; ^1^H-NMR (CDCl_3_) δ 9.39 (s, 1H, NH), 8.43 (dd, *J* = 8.0, 1.4 Hz, 1H, Ar-H), 7.89 (s, 1H, pyrazole-H), 7.36–6.96 (m, 3H, Ar-H), 7.10 (t, 1H, CF_2_H), 3.90 (s, 3H, CH_3_), 2.98–2.69 (m, 2H, CH_2_), 2.56 (s, 3H, CH_3_), 1.37 (dt, *J* = 14.6, 6.9 Hz, 2H, CH_2_), 1.23 (dq, *J* = 14.3, 7.1 Hz, 2H, CH_2_), 0.80 (t, *J* = 7.2 Hz, 3H, CH_3_). ^13^C-NMR (CDCl_3_) δ 159.14, 141.96, 134.57, 133.52, 125.06, 123.66, 121.52, 119.38, 117.45, 110.60, 56.39, 43.35, 39.30, 29.51, 20.20, 13.65. HRMS (ESI), *m/z* calcd for C_17_H_22_F_2_N_4_O (M+H)^+^ 337.1834, found 337.1837.

*N-(2-(Diethylamino)phenyl)-3-(difluoromethyl)-1-methyl-1H-pyrazole-4-carboxamide* (**9l**): White powder; yield 79%; mp 85–86 °C; ^1^H-NMR (CDCl_3_) δ 9.56 (s, 1H, NH), 8.44 (d, *J* = 7.9 Hz, 1H, Ar-H), 7.88 (s, 1H, pyrazole-H), 7.34–6.95 (m, 3H, Ar-H), 7.12 (t, 1H, CF_2_H) ,3.86 (d, *J* = 6.5 Hz, 3H, CH_3_), 2.89 (q, *J* = 7.1 Hz, 4H, CH_2_), 0.87 (t, *J* = 7.1 Hz, 6H, CH_3_). ^13^C-NMR (CDCl_3_) δ 159.02, 143.70, 139.05, 136.41, 133.48, 125.36, 123.44, 123.07, 119.03, 117.45, 110.62, 48.98, 39.29, 12.10. HRMS (ESI), *m/z* calcd for C_16_H_20_F_2_N_4_O (M+H)^+^ 323.1678, found 323.1677.

*N-(2-(5-bromo-1H-indazol-1-yl)phenyl)-3-(difluoromethyl)-1-methyl-1H-pyrazole-4-carboxamide* (**9m**): White crystals; yield 74%; mp 90–92 °C; ^1^H-NMR (CDCl_3_) δ 9.62 (s, 1H, NH), 8.43 (t, *J* = 7.3 Hz, 1H, Ar-H), 8.24(s, 1H, pyrazole-H), 7.89–7.58 (m, 2H, Ar-H), 7.49 (dd, *J* = 13.1, 4.9 Hz, 2H, Ar-H), 7.34 (dt, *J* = 8.9, 6.2 Hz, 2H, Ar-H), 7.20 (t, 1H, CF_2_H) , 7.27–7.02 (m, 1H, Ar-H), 3.85 (s, 3H, CH_3_). ^13^C-NMR (CDCl_3_) δ 159.22, 139.76, 135.76, 132.17, 131.46, 128.57, 128.01, 127.82, 124.36, 124.21, 123.52, 123.31, 122.02, 121.18, 110.48, 109.59, 39.44. HRMS (ESI), *m/z* calcd for C_19_H_14_BrF_2_N_5_O (M+H)^+^ 446.0423, found 446.0423.

*N-(2-(6-Bromo-5-methyl-1H-indazol-1-yl)phenyl)-3-(difluoromethyl)-1-methyl-1H-pyrazole-4-carbox- amide* (**9n**): White crystals; yield 69%; mp 150–152 °C; ^1^H-NMR (CDCl_3_) δ 9.88 (s, 1H, NH), 8.63–8.39 (m, 1H, Ar-H), 8.21 (s, 1H, pyrazole-H), 7.67 (s, 1H, Ar-H), 7.53 (d, *J* = 7.4 Hz, 1H, Ar-H), 7.52–7.37 (m, 3H, Ar-H), 7.29–7.20 (m, 2H, Ar-H), 7.09 (t, 1H, CF_2_H), 3.88 (s, 3H, CH_3_), 2.45 (s, 3H, CH_3_). ^13^C-NMR (CDCl_3_) δ 159.23, 138.47, 135.15, 132.11, 131.67, 131.37, 129.92, 128.69, 127.83, 124.67, 124.14, 124.00, 123.23, 120.13, 116.93, 110.20, 109.55, 39.44, 21.00. HRMS (ESI), *m/z* calcd for C_20_H_16_BrF_2_N_5_O (M+H)^+^ 460.0579, found 460.0573.

### 3.3. Bioassays

The fungi were provided by the Laboratory of Institute of Plant Protection, Chinese Academy of Agricultural Sciences (Beijing, China). The fungicidal activity of the target compounds was tested *in vitro* against the seven plant pathogenic fungi using the mycelia growth inhibition method [15]. The tested compounds were dissolved in DMSO at a concentration of 10 mg·mL^−1^. The media containing compounds at a concentration of 50 μg·mL^−1^ were then poured into Petri dishes for initial screening. In the precision antifungal test, the 10 mg·mL^−1^ solution was diluted to 100, 50, 25, 12.5, 6.25, 3.125, 1.56 μg·mL^−1^ and the above experiments were repeated three times, the inhibition rates were calculated separately. The statistical analyses were performed by SPSS software version 17.0.

### 3.4. QSAR Analyses

Topomer CoMFA (in the SYBYL X 2.0 program) was performed to analysis the relationship between structure and activity. Topomer CoMFA is an alignment-independent 3D-QSAR method that combines the topomer search method [16] with the conventional CoMFA method. Besides the core of the molecule, we split the functional of compound into two R-groups that refer to the R_1_ (amide moiety) and R_2_ (carboxylic acid moiety) groups. In total, 21 compounds obtained from synthesis were used to create a data set in which the inhibition rate of all compounds was determined (Table 1) against *Colletotrichum orbiculare*. Three-dimensional structures of the target compounds were built by the Chem3D software version 12.0.

### 3.5. Molecular Docking

Docking was performed by Surflex-Dock (in the SYBYL X2.0 program). The ligand structures were energetically minimized using MM2 energy minimizations in ChemBio 3D. All bound water and key ligands were eliminated from the protein, and the polar hydrogen atoms and the AMBER7 FF99 charges were added to the proteins.

## 4. Conclusions

Twenty-one novel 3-(difluoromethyl)-1-methyl-1*H*-pyrazole-4-carboxylic acid amides had been synthesized by introducing the 3-(difluoromethyl)-1-methyl-1*H*-pyrazole-4-acyl group in these amide compounds. The bioassays showed that all of them exhibited moderate to remarkable antifungal activities against the seven tested fungi. The compounds containing indazole groups displayed stronger antifungal activities than the others. By comparing the activities and structures of the compounds **9c** and **9m** with the highly antifungal compounds which we found in previous work [2], it could be determined that the indazole group plays a significant role in improving the activities of the molecules. We found a novel lead compound **9m** with higher antifungal activity and broader spectrum than the control boscalid. Topomer CoMFA was employed to develop a 3D QSAR model on the antifungal activity of target molecules. A molecular docking study showed the mode of action of these structures on SDH. The present findings provided a powerful complement to the SDHIs of fungicides, and warrant future investigation of the mechanism of action of these analogues. Further studies on biological behavior and structural optimization are in progress in our laboratory.
